# Supplementary material for: Translational initiation factor eIF5 replaces eIF1 on the 40S ribosomal subunit to promote start-codon recognition
Source: eLife. 2018 Nov 30;7:e39273. doi: 10.7554/eLife.39273 (PMC6298780; doi:10.7554/eLife.39273)
Supplement: Supplementary file 1. [file elife-39273-supp1.docx]

**Table S1. Plasmids used in this study**

| **Plasmid** | **Description** | **Source** |
| --- | --- | --- |
| YCplac111 | sc *LEU2* cloning vector | (Gietz and Sugino 1988) |
| YCplac22 | sc *TRP1* cloning vector | (Gietz and Sugino 1988) |
| pAS5-101 | sc *LEU2 TIF5-FL* in YCplac111 | (Saini et al. 2014) |
| pSK5-608 | sc *LEU2 TIF5-G29R-FL* in YCplac111 | This study |
| pSK5-736 | sc *LEU2*  *TIF5*-E26K*-FL* in YCplac111 | This study |
| pSK5-737 | sc *LEU2*  *TIF5-R28A-FL* in YCplac111 | This study |
| pSK5-738 | sc *LEU2 TIF5-R28E-FL* in YCplac111 | This study |
| pSK5-739 | sc *LEU2 TIF5-G29E-FL* in YCplac111 | This study |
| pSK5-741 | sc *LEU2*  *TIF5-K55E-FL* in YCplac111 | This study |
| pSK5-742 | sc *LEU2* *TIF5-N30A-FL* in YCplac111 | This study |
| pSK5-743 | sc *LEU2 TIF5-N30E-FL* in YCplac111 | This study |
| pSK5-750 | sc *LEU2 TIF5-R73A-FL* in YCplac111 | This study |
| pSK5-835 | sc *LEU2 TIF5-N30R-FL* in YCplac111 | This study |
| pSK5-840 | sc *LEU2 TIF5*-*K142E*-*FL* in YCplac111 | This study |
| pSKY-504 | sc *LEU2 TIF5-G78R-FL* in YCplac111 | This study |
| YCpSUI3-2 | sc *TRP1 SUI3-S264Y* in YCplac22 | (Valasek et al. 2004) |
| p367 | sc *URA3* *HIS4(ATG)-lacZ* | (Donahue and Cigan 1988) |
| p391 | sc *URA3 HIS4(TTG)-lacZ* | (Donahue and Cigan 1988) |
| p3342 | sc *URA3 TIF5* in YCplac33 | (Asano et al. 1999) |
| pJCB101 | sc *LEU2 SUI1* in YCplac111 | (Martin-Marcos et al. 2011) |
| pPMB03 | sc *sui1-L96P* in YCplac111 | (Martin-Marcos et al. 2011) |
