# Supplementary material for: Translational initiation factor eIF5 replaces eIF1 on the 40S ribosomal subunit to promote start-codon recognition
Source: eLife. 2018 Nov 30;7:e39273. doi: 10.7554/eLife.39273 (PMC6298780; doi:10.7554/eLife.39273)
Supplement: Supplementary file 2. [file elife-39273-supp2.docx]

**Table S2. Yeast strains used in this study**

| **Strains** | **Description** | **Source** |
| --- | --- | --- |
| ASY100 | *MATα ura3-52 trp1Δ63 leu2-112 his4-301 (ACG) tif5Δ::kanMX* p3342 [*TIF5, URA3*] | (Saini et al. 2014) |
| ASY101 | *MATα ura3-52 trp1Δ63 leu2-112 his4-301 (ACG) tif5Δ::kanMX* pAS5-101 [*TIF5-FL, LEU2*] | (Saini et al. 2014) |
| SKY630 | *MATα ura3-52 trp1Δ63 leu2-112 his4-301 (ACG) tif5Δ::kanMX* pSK5-608 *[TIF5-G29R-FL, LEU2]* | This study |
| SKY754 | *MATα ura3-52 trp1Δ63 leu2-112 his4-301 (ACG) tif5Δ::kanMX* pSK5-736 [*TIF5-E26K-FL, LEU2*] | This study |
| SKY755 | *MATα ura3-52 trp1Δ63 leu2-112 his4-301 (ACG) tif5Δ::kanMX* pSK5-737 [*TIF5-R28A-FL, LEU2*] | This study |
| SKY756 | *MATα ura3-52 trp1Δ63 leu2-112 his4-301 (ACG) tif5Δ::kanMX* pSK5-738 [*TIF5-R28E-FL, LEU2*] | This study |
| SKY757 | *MATα ura3-52 trp1Δ63 leu2-112 his4-301 (ACG) tif5Δ::kanMX* pSK5-739 [*TIF5-G29E-FL, LEU2*] | This study |
| SKY759 | *MATα ura3-52 trp1Δ63 leu2-112 his4-301 (ACG) tif5Δ::kanMX* pSK5-741 [*TIF5-K55E-FL, LEU2*] | This study |
| SKY760 | *MATα ura3-52 trp1Δ63 leu2-112 his4-301 (ACG) tif5Δ::kanMX* pSK5-742 [*TIF5-N30A-FL, LEU2*] | This study |
| SKY761 | *MATα ura3-52 trp1Δ63 leu2-112 his4-301 (ACG) tif5Δ::kanMX* pSK5-743 [*TIF5-N30E-FL, LEU2*] | This study |
| SKY768 | *MATα ura3-52 trp1Δ63 leu2-112 his4-301 (ACG) tif5Δ::kanMX* pSK5-750 [*TIF5-R73A-FL, LEU2*] | This study |
| SKY844 | *MATα ura3-52 trp1Δ63 leu2-112 his4-301 (ACG) tif5Δ::kanMX* pSK5-835 [*TIF5-* *N30R-FL, LEU2*] | This study |
| SKY867 | *MATα ura3-52 trp1Δ63 leu2-112 his4-301 (ACG) tif5Δ::kanMX* pSK5-840 [*TIF5-* *K142E-FL, LEU2*] | This study |
| PMY30 | *MATα ura3-52 trp1Δ63 leu2-112 his4-301 (ACG) sui1Δ::hisG* pJCB101 [ sc *LEU2 SUI1*] | (Martin-Marcos et al. 2011) |
| PMY33 | *MATα ura3-52 trp1Δ63 leu2-112 his4-301 (ACG) sui1Δ::hisG* pPMB03 [ sc *LEU2 sui1-L96P*] | (Martin-Marcos et al. 2011) |
